# Residue of Chlormequat and Regulatory Effects on the Specialized Metabolites of Astragali Radix

**DOI:** 10.3390/molecules28196754

**Published:** 2023-09-22

**Authors:** Honghan Qin, Lei Xie, Yimei Zang, Jia Han, Jing Yu, Zuliang Luo, Xiaojun Ma

**Affiliations:** 1 College of Pharmacy, Youjiang Medical University for Nationalities, Baise 533000, China; qinhonghanyyxb@126.com; 2 Institute of Medicinal Plant Development, Chinese Academy of Medical Sciences, Peking Union Medical College, Beijing 100193, China; 3 Biomedicine College, Beijing City University, Beijing 100094, China; 4 Yunnan Key Laboratory of Southern Medicinal Utilization, Yunnan Branch Institute of Medicinal Plant Development, Chinese Academy of Medical Sciences, Peking Union Medical College, Jinghong 666100, China

**Keywords:** astragali radix, chlormequat, residue, regulatory effects, specialized metabolites

## Abstract

Presently, the utilization of chlormequat in *Astragalus mongholicus* Bunge (Leguminosae) cultivation is prevalent for augmenting rhizome (Astragali Radix) yield. However, indiscriminate and excessive chlormequat employment can detrimentally influence Astragali Radix quality and safety. This research aimed to comprehensively comprehend chlormequat risks and its influence on Astragali Radix metabolites. Diverse chlormequat concentrations were employed in *Astragalus mongholicus* cultivation, with subsequent analysis of residual chlormequat levels in Astragali Radix across treatment groups. Astragali Radix metabolic profiling was conducted through UPLC-QTOF-MS, and thirteen principal active components were quantified via UFLC-MS/MS. Findings revealed a direct correlation between chlormequat residue levels in Astragali Radix and application concentration, with high-dose residue surpassing 5.0 mg/kg. Metabolomics analysis identified twenty-six distinct saponin and flavonoid metabolites. Notably, the application of chlormequat led to the upregulation of seven saponins (e.g., astragaloside I and II) and downregulation of six flavonoids (e.g., methylnissolin-3-*O*-glucoside and astraisoflavan-7-*O*-β-d-glucoside). Quantitative analysis demonstrated variable contents of active ingredients due to differing chlormequat concentrations, leading to astragaloside I increase (14.59–62.55%) and isoastragaloside II increase (4.8–55.63%), while methylnissolin-3-*O*-glucoside decreased (22.18–41.69%), as did astraisoflavan-7-*O*-β-d-glucoside (21.09–47.78%). In conclusion, chlormequat application influenced multiple active components in Astragali Radix, causing constituent proportion variations. Elevated chlormequat concentrations led to increased active components alongside heightened chlormequat residues in Astragali Radix. Consequently, prudent chlormequat application during Astragali Radix production is imperative to avert potential detriments to its quality and safety.

## 1. Introduction

Astragali Radix, the dry roots of *Astragalus membranaceus* (Fisch.) Bunge or *Astragalus mongholicus* Bunge (Family Leguminosae), is among the most extensively utilized Chinese herbal medicines for Qi tonification, referring to the practice of nourishing and fortifying the body’s vital energy [1]. Astragali Radix is a frequently used medicinal herb in clinical practice. It belongs to the category of “food-medicine homology” and is renowned not only as a supreme Qi-tonifying herb but also for its diuretic and wound-healing effects [2]. It is frequently used in the treatment of clinical conditions such as fatigue resulting from Qi deficiency, persistent diarrhea, and rectal prolapse [3,4,5]. As technological progress continues, Astragali Radix has expanded its utility beyond pharmaceuticals, extending to health products, cosmetics, and associated domains, frequently integrated into offerings designed to fortify the body’s immune system [6]. The intricate composition of Astragali Radix encompasses a variety of chemical constituents, notably saponins and flavonoids, alongside polysaccharides, alkaloids, and trace elements [7]. These constituents collectively contribute to a diverse array of pharmacological effects exhibited by Astragali Radix, encompassing immune modulation, safeguarding cardiovascular health, eliciting anti-tumor responses, providing hepatoprotection, facilitating kidney preservation, and more [8,9].

*Astragalus mongholicus* is the main source of Astragali Radix and is mainly grown in regions like Gansu and Inner Mongolia in China [10]. Gansu, in particular, accounts for over 60% of the national production. Recently, there has been increased interest in the medicinal properties of Astragali Radix, leading to a shortage in the market. In Gansu’s Dingxi City, where *Astragalus mongholicus* is a major crop, farmers often use chlormequat, a common plant growth regulator, to manage plant growth [11,12]. Chlormequat helps control plant height, encourages compact growth, and prevents lodging. This results in improved photosynthesis, making the crop more resistant to drought and cold, ultimately increasing yield and profit. Applying chlormequat to *Astragalus mongholicus* also helps stimulate root growth, shortens the cultivation time, and boosts the yield and profit of Astragali Radix [13,14]. However, using too much or using it improperly can change the herb’s characteristics and active components. Therefore, addressing concerns about chlormequat residues in Astragali Radix and how they affect its main active ingredients is a crucial scientific issue that needs attention.

Medicinal herbs are unique because their safety, effectiveness, and healing properties are closely linked. Chlormequat, a substance used to help plants grow, can be toxic. Therefore, when using it with medicinal plants, we need to be careful about safety and its impact on the plant’s beneficial compounds. Therefore, it is crucial to conduct tests before using chlormequat in the cultivation of medicinal plants [15]. Research has shown that chlormequat can have different effects on the active ingredients in medicinal plants. For example, it can reduce certain compounds in the roots of *Scutellaria baicalensis* (Lamiaceae) but increase others in its above-ground parts [16]. Similarly, it can boost certain compounds in *Polygonatum odoratum* (Asparagaceae) [17]. However, we do not yet know how chlormequat affects the active ingredients in Astragali Radix. With this in mind, our current study aims to test how different amounts of chlormequat impact Astragali Radix. Our main goal is to check if it is safe and scientifically sound to use chlormequat when growing *Astragalus mongholicus*. Ultimately, we want to lay the groundwork for standardized Astragali Radix cultivation in the future.

In this study, a comprehensive investigation was conducted concerning the residual presence of chlormequat in Astragali Radix following its application, along with its impact on the herb’s specialized metabolites, especially saponins and flavonoids. Initially, a qualitative assessment was executed using UPLC-QTOF-MS to scrutinize the specialized metabolites within Astragali Radix treated with varying chlormequat concentrations. This analytical approach unveiled distinct regulatory patterns exerted by chlormequat on the accumulation of saponins and flavonoids, thereby identifying unique metabolic markers directly associated with the influence of chlormequat. Then, UFLC-MS/MS was employed for a quantitative evaluation encompassing the thirteen main active components present in Astragali Radix. Through this methodology, the study systematically delved into the regulatory dynamics of chlormequat on the specialized metabolites of Astragali Radix throughout its growth cycle. This research endeavor contributes a robust scientific framework to guide the utilization of chlormequat in *Astragalus mongholicus* cultivation, ultimately enriching our understanding to bolster the herb’s quality and ensure its clinical safety.

## 2. Results and Discussion

### 2.1. Residue of Chlormequat in Astragali Radix

**Figure 1:**
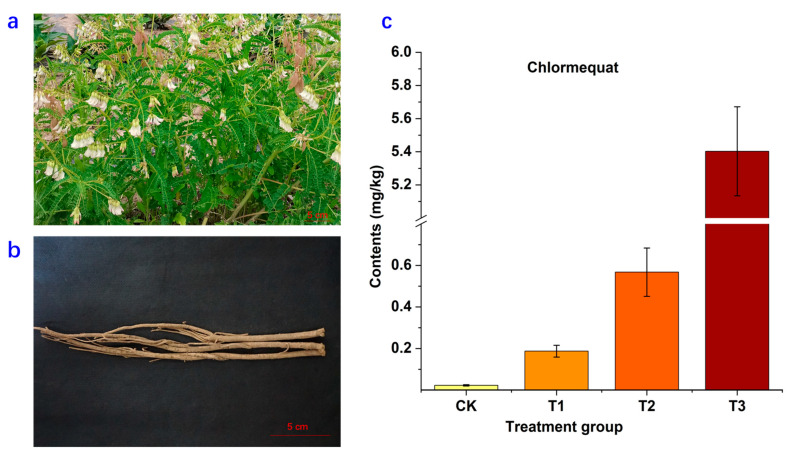
The plant morphology ((**a**) aerial part of *Astragalus mongholicus*; (**b**) rhizome) of *Astragalus mongholicus* and chlormequat residue in different treatment groups of Astragali Radix (**c**). CK represents the control group; T1 indicates the chlormequat concentration was 0.1 g/L; T2 indicates the chlormequat concentration was 1.0 g/L; and T3 indicates the chlormequat concentration was 10.0 g/L.

The analysis of chlormequat residue in Astragali Radix was performed using LC-MS/MS, as outlined in previously established methodologies [18]. The results illuminated the presence of chlormequat residue in Astragali Radix, with the levels of residue demonstrating a positive correlation with the applied concentration (Figure 1 and Supplementary Table S1). Specifically, in instances where the chlormequat concentration was 0.1 g/L (T1), the residue in Astragali Radix exceeded 0.1 mg/kg. At higher concentrations of 1.0 g/L (T2) and 10.0 g/L (T3), the residues surpassed 0.5 mg/kg and 5.0 mg/kg, respectively.

While explicit standards for the maximum residue limit (MRL) of chlormequat in traditional Chinese medicine are not currently defined, the national food safety guidelines for maximum pesticide residues in food offer relevant insights [19]. The stipulated residual limit for various grains and oilseeds ranges from 0.1 to 10.0 mg/kg. Among the listed food categories (excluding oats), the maximum residual limit remains below 5.0 mg/kg. However, the residues detected in this investigation consistently exceeded 5.0 mg/kg after the application of higher chlormequat concentrations (10.0 g/L). Consequently, the recurrent application of high concentrations during *Astragalus mongholicus* cultivation could culminate in substantial residue accumulation in Astragali Radix, potentially compromising the local ecological balance. Significantly, Astragali Radix operates within the domain of food–medicine homology, heightening the importance of managing residue levels judiciously. Excessive residue may inevitably prompt alterations in the herb’s therapeutic efficacy and even pose potential hazards to human health.

Presently, a growing body of research indicates potential effects of chlormequat on mammalian reproduction. Experimental evidence showed that chlormequat might restrain the puberty onset and impair the reproductive functions in male rats through the HPT axis and disturb the sperm motility through PP1γ2 [20,21]. Notably, Xiagedeer et al. have demonstrated that maternal exposure to chlormequat chloride at an acceptable daily intake (ADI) level during pregnancy disrupts normal embryo growth processes by elevating embryonic growth hormone levels [22,23].

Our earlier extensive sample analysis of traditional Chinese medicinal materials revealed the widespread presence of chlormequat, particularly in root and rhizome-based medicinal plants [18]. This indicates the common utilization of chlormequat in the cultivation of traditional Chinese medicinal materials. The findings from this study distinctly reveal elevated residual levels of chlormequat in Astragalus Radix subsequent to its application. Given the significance of Astragalus Radix as a crucial component in traditional Chinese medicine, the lingering presence of chlormequat residues holds the potential to jeopardize the health of users of traditional herbal remedies. Consequently, the utilization of chlormequat in *Astragalus mongholicus* demands a heightened focus on safety considerations.

### 2.2. Metabolite Changes of Astragali Radix

**Figure 2:**
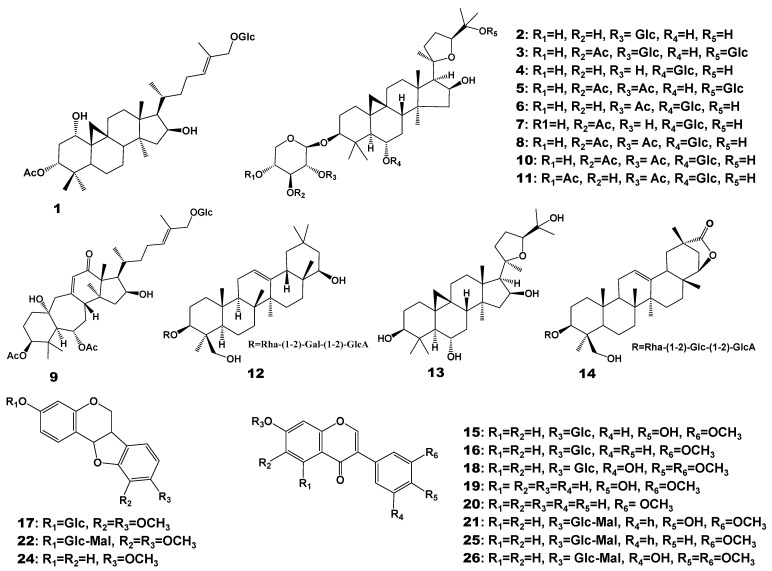
The chemical structures of compounds (**1**–**22**, **24**–**26**) identified in Astragali Radix. Glc: β-d-Glucose, GlcA: β-d-Glucuronic acid, Rha: α-L-rhamnose, Gal: β-d-Galactose, Ac: acetic acid, Mal: Malonic acid.

The metabolites of Astragalus Radix were analyzed by UPLC-QTOF-MS using an ESI ion source. According to existing literature, the main metabolites of Radix Astragali, especially saponins, can present richer characteristic ion fragments in positive ion mode [24,25]. Therefore, in this study, positive ion mode was chosen for sample determination and analysis. The total ion chromatograms (TIC) in positive ion modes of Radix Astragali are presented in Supplementary Figure S1. The reference compounds were initially analyzed to obtain the retention time and characteristic fragmentation pathway data prior to observing the samples. We then identified the compounds of Astragali Radix by comparing the retention time and mass spectra data with those of the standards and the literature data [26,27,28]. Notably, the mass spectrometry of both standards and samples uniformly demonstrated that the precursor ions for various substances were [M + H]^+^. Compound identification was conducted based on both the precursor ions and MS/MS fragments. This integrated approach culminated in the initial identification of fourteen saponins and twelve flavonoid compounds within Astragalus Radix. These specific compounds and their details are cataloged in Table 1, and the chemical structures of compounds (**1**–**22**, **24**–**26**) identified are shown in Figure 2. The accurate structure of compound **23** is unknown, therefore its structure is not depicted in Figure 2.

The chemical composition of Astragalus Radix mainly includes saponins and flavonoid compounds. Flavonoid compounds can be classified into four major categories: flavonoids, isoflavonoids, isoflavanes, and pterocarpans; while saponin compounds include cycloartane-type saponins, oleanane-type saponins, and soybean saponins. The saponin components we identified encompass nine cycloartane-type triterpenoids and three other triterpenoids. The twelve identified flavonoids mainly belong to isoflavonoids. In previous research, these components have consistently shown higher levels of presence in Astragalus Radix, making them the most focused-upon chemical constituents. Furthermore, studies related to the bioactivity of Astragalus Radix primarily revolve around these two main categories of components [7,29,30].

**Figure 3:**
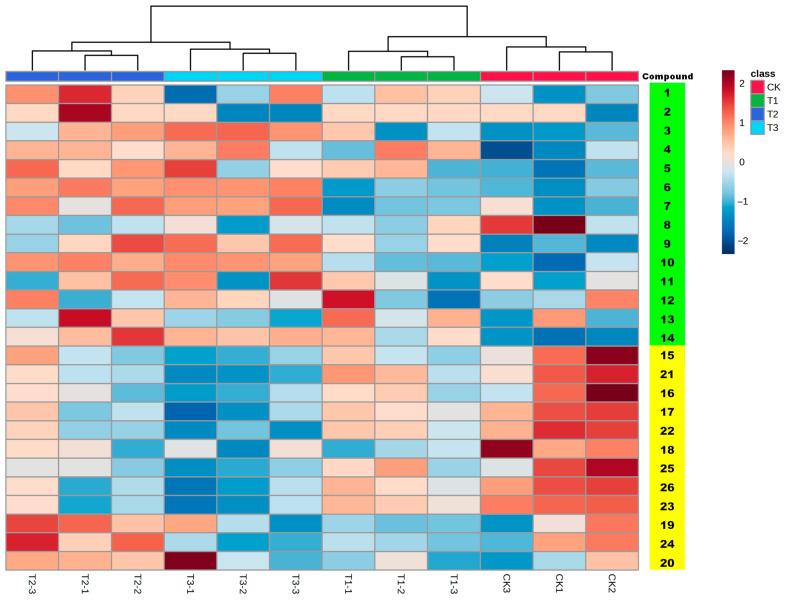
Analysis of metabolites changes in different treatment groups of Astragali Radix. The metabolites under the green background represent saponins, the metabolites under the yellow background represent flavonoids.

To directly visualize the patterns of metabolic alterations in Astragali Radix following chlormequat treatment, a data matrix was constructed utilizing identified metabolites and their corresponding response intensities across various treatment samples (Supplementary Table S2). Subsequently, this data matrix underwent cluster analysis and heatmap generation via the web version of MetaboAnalyst 5.0 (https://www.metaboanalyst.ca/, accessed on 10 March 2023), yielding the heatmap displayed in Figure 3. The heatmap showed that the metabolites can be grouped into two subgroups, with similar trends in metabolic content changes. In the saponins subgroup, the majority of metabolites show an upregulation trend after chlormequat treatment. On the other hand, in the flavonoid subgroup, most metabolites exhibit a downregulation trend.

One-way ANOVA was performed on statistical analysis for the experimental results. Based on the criterion of *p*-value (0.05), thirteen differential metabolites were found, as shown in Table 1. Within these thirteen metabolites, in comparison to the blank control group, the content of seven saponin metabolites exhibited significant upregulation. Notable among these are astragaloside I, astragaloside II, isoastragaloside II, astragaloside III, astragaloside V, huangqiyenin G, and astroolesaponin D. Concurrently, six flavonoid metabolites experienced significant downregulation, including astrapterocarpanglucoside-6″-malonate, methylnissolin-3-*O*-glucoside, astraisoflavan-7-*O*-β-d-glucoside, calycosin-7-glucoside-6″-malonate, and methylnissolin-3-*O*-glucoside-6″-malonate. This discernible pattern suggests that chlormequat might contribute to the augmentation of astragaloside content while concurrently exerting a suppressive influence on the accumulation of flavonoid components in Astragali Radix. Importantly, it is noteworthy that distinct components exhibit diverse regulatory trends in response to chlormequat.

With the increasing demand for natural secondary metabolites in the market in recent years, various research strategies have been extensively employed to enhance the production of plant secondary metabolites. Plant growth regulators (PGRs) have been used as effective inducers to stimulate the production of plant secondary metabolites. Numerous studies have been conducted in a wide range of plants to investigate and confirm the impact of different PGRs on the generation of secondary metabolites. In 2018, Jamwal et al. summarized the regulatory effects of different PGRs on secondary metabolites in various medicinal plants [31]. Research has indicated that different PGRs, whether used alone or in combination, can significantly influence the accumulation of secondary metabolites such as terpenes, coumarins, flavonoids, isoflavones, and alkaloids. Chlormequat’s regulatory effects on the active constituents of medicinal plants like *Scutellaria baicalensis* (Lamiaceae), *Lonicera japonica* (Caprifoliaceae), *Ginkgo biloba* (Ginkgoaceae), *Hypericum perforatum* (Hypericaceae), and *Bupleurum chinense* (Umbelliferae) have been found to vary. It has been observed that chlormequat can upregulate lignin in *S. baicalensis* and saponins in *B. chinense*, while downregulating flavonoids in *S. baicalensis* and *L. japonica* [11,32]. The results of this study indicate that the effects of chlormequat on saponin and flavonoid components are consistent with previous relevant research, once again confirming the regulatory role of chlormequat in the secondary metabolites of medicinal plants.

### 2.3. Quantitative Analysis of Thirteen Active Compounds in Astragali Radix

#### 2.3.1. Optimization of UFLC-MS/MS Condition

To achieve optimal analytical conditions for the target compounds within Astragalus Radix, systematic optimization of chromatographic conditions and mass spectrometric parameters was conducted. The mobile phase composition holds significant influence over ionization efficiency and analyte separation. Through reference to prior investigations [33,34], the most suitable mobile phase composition and chromatographic conditions were identified to ensure optimal peak-shape response intensity and resolution. Detailed information regarding the optimal chromatographic conditions can be found in the Materials and Methods section.

**Figure 4:**
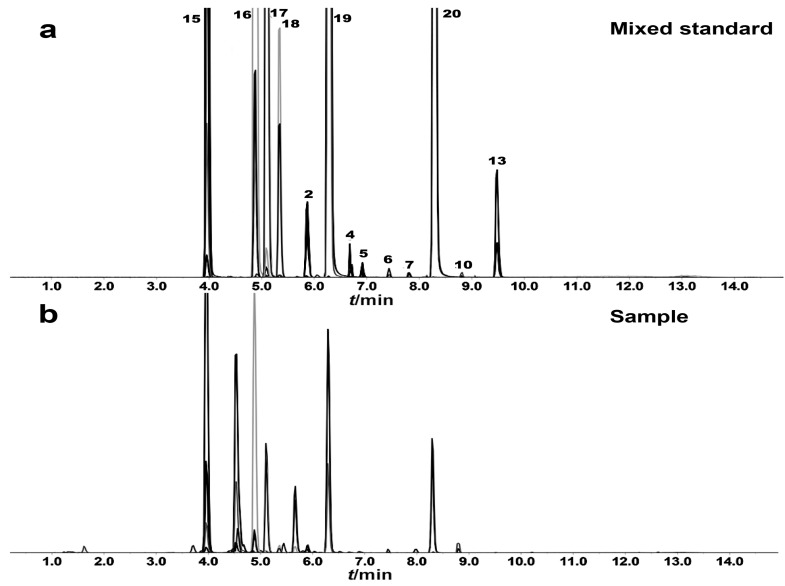
Multiple reaction monitoring (MRM) chromatograms of the mixed standard (**a**) and Astragali Radix sample (**b**). The numbers represent compound numbers. **15**, Calycosin-7-glucoside; **16**, Ononin; **17**, Methylnissolin 3-*O*-glucoside; **18**, Astraisoflavan-7-*O*-β-d-glucoside; **2**, Astragaloside III; **19**, Calycosin; **4**, Astragaloside IV; **5**, Isoastragaloside IV; **6**, Astragaloside II; **7**, Isoastragaloside II; **20**, Formononetin; **10**, Astragaloside I; **13**, Cycloastragenol.

To determine the optimal mass spectrometric behaviors and parameters for the identification and quantification of the targeted analytes, a standard solution (100 ng/mL) of each analyte was directly introduced into the mass spectrometer using a syringe pump (Harvard apparatus, South Natick, MA, USA) at a flow rate of 10 µL/min. The significant mass spectrometry parameter declustering potential (DP) and collision energies (CEs) were optimized to achieve maximum sensitivity. Multiple reaction monitoring (MRM) scanning mode was selected for quantification. The optimized MRM transitions and their corresponding parameters are provided in Supplementary Table S3, with representative chromatograms of the targeted analytes shown in Figure 4.

#### 2.3.2. Method Validation

The optimized UFLC-MS/MS method for quantitative analysis was subjected to comprehensive validation, encompassing assessments of linearity, limits of detection (LODs), limits of quantification (LOQs), precision, stability, and recovery of the targeted analytes. The detailed results are provided in Supplementary Table S4. The calibration curves exhibited robust linearity, as evidenced by correlation coefficients (r^2^) ranging from 0.9925 to 0.9998 across all analytes within the concentration ranges. The LODs and LOQs spanning 0.2–20 and 0.5–50 ng/mL, respectively, underscore the heightened sensitivity of the developed method. Precision evaluation employed the relative standard deviation (RSD), with intra-day precision ranging from 1.01% to 2.65%, and inter-day precision between 0.97% and 3.81%, establishing the method’s reliability. The stability analysis demonstrated that all analytes exhibited favorable stability, indicated by an RSD for a peak area of less than 3.81% over a span of 48 h. Recovery rates across all analytes ranged from 81.54% to 110.49%, accompanied by RSD values spanning 0.34% to 5.36%. These results collectively affirm the method’s sound reliability, precision, and accuracy.

#### 2.3.3. Sample Analysis

**Figure 5:**
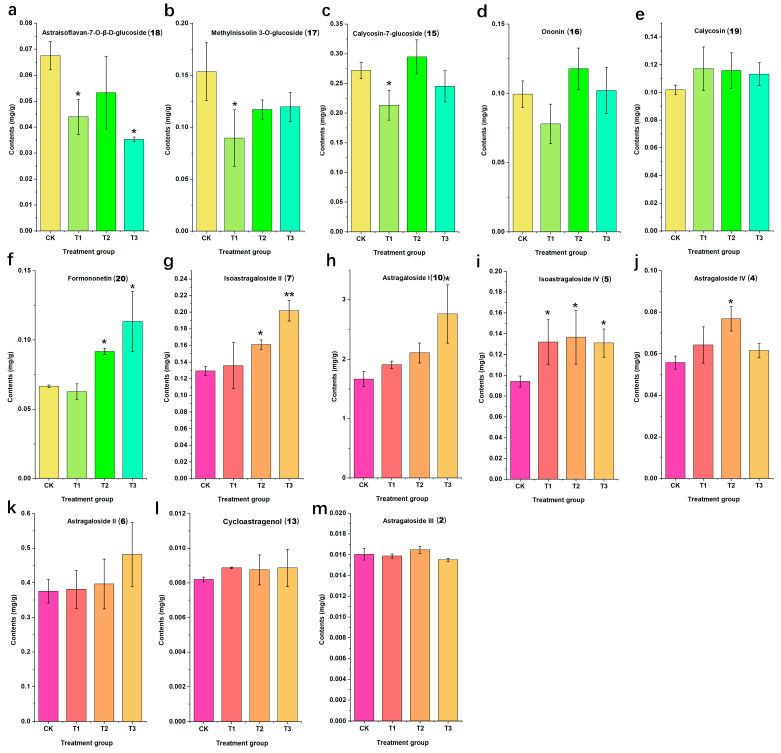
The contents of thirteen compounds in Astragali Radix from different groups. The asterisk represents a statistically significant difference (*p* < 0.05). Astraisoflavan-7-*O*-β-d-glucoside (**a**); Methylnissolin 3-*O*-glucoside (**b**); Calycosin-7-glucoside (**c**); Ononin (**d**); Calycosin (**e**); Formononetin (**f**); Isoastragaloside II (**g**); Astragaloside I (**h**); Isoastragaloside IV (**i**); Astragaloside IV (**j**); Astragaloside II (**k**); Cycloastragenol (**l**); Astragaloside III (**m**). CK represents the control group; T1 indicates the chlormequat concentration was 0.1 g/L; T2 indicates the chlormequat concentration was 1.0 g/L; and T3 indicates the chlormequat concentration was 10.0 g/L. The numbers in parentheses represent compound numbers; (**a**–**f**) are flavonoid components, and (**h**–**m**) are saponin components. The asterisk denotes statistically significant differences * *p* < 0.05; ** *p* < 0.01. Among the six measured flavonoid components, calycosin-7-glucoside exhibits the highest content, followed by methylnissolin 3-*O*-glucoside, calycosin, ononin, formononetin, and astraisoflavan-7-*O*-β-d-glucoside. As depicted in Figure 5, the application of varying chlormequat concentrations notably diminishes the content of methylnissolin 3-*O*-glucoside, astraisoflavan-7-*O*-β-d-glucoside, and calycosin-7-glucoside (*p* < 0.05). In comparison to the control group, distinct chlormequat concentrations correspond to a decline in methylnissolin 3-*O*-glucoside content ranging from 22.18% to 41.69%, and a reduction in astraisoflavan-7-*O*-β-d-glucoside content ranging from 21.09% to 47.78%. Furthermore, diverse concentrations of chlormequat application significantly augment the content of formononetin, manifesting an increase ranging from 5.81% to 70.08%. Nonetheless, the content of ononin and calycosin remains unaffected by the application of varying chlormequat concentrations.

The results of content determination for Astragali Radix samples are presented in Table 2 and Figure 5. The results reveal that astragaloside I was the highest content among the thirteen target analytes. Specifically, the content of seven astragaloside components follows the order of astragaloside I, astragaloside II, isoastragaloside II, isoastragaloside IV, astragaloside IV, astragaloside III, and cycloastragenol in decreasing magnitudes. The data represented in Figure 5 highlights that the application of elevated chlormequat concentrations significantly stimulates the accumulation of astragaloside I, astragaloside IV, isoastragaloside II, and isoastragaloside IV (*p* < 0.05). Relative to the control group, different chlormequat concentrations prompted varying degrees of increase in astragaloside I content, ranging from 14.59% to 62.55%. Similarly, isoastragaloside II experienced an increase ranging from 4.8% to 55.63%, and isoastragaloside IV exhibited an increase ranging from 39.40% to 45.34%. However, the content of astragaloside II, astragaloside III, and cycloastragenol showed no significant alteration upon the application of diverse chlormequat concentrations.

The quantitative results demonstrate that the application of various concentrations of chlormequat leads to an augmentation in the content of four types of saponins (astragaloside I, isoastragaloside II, astragaloside IV, and isoastragaloside IV) and one flavonoid compound (formononetin) in Astragali Radix. These findings underscore the notable positive regulatory role of chlormequat in the accumulation of saponin components. Conversely, a significant inverse regulatory effect of chlormequat is observed on the content of three flavonoid compounds (methylnissolin 3-*O*-glucoside, astraisoflavan-7-*O*-β-d-glucoside, and calycosin-7-glucoside) leading to their substantial reduction. Notably, the concentration of chlormequat exhibits a strong correlation with its regulatory effect on related active components.

Bioactivity-related research findings indicate that astragaloside I, isoastragaloside II, astragaloside IV, and isoastragaloside IV play essential roles in cardiovascular protection, liver protection, anti-liver fibrosis, anti-cancer, anti-inflammatory, neuroprotection, and immunomodulatory activity [28,35,36,37,38,39]. Methylnissolin 3-*O*-glucoside, astraisoflavan-7-*O*-β-d-glucoside, and calycosin-7-glucoside exhibit activities such as cardiovascular protective effects, neuroprotection, anti-inflammatory, and immunomodulatory activity [40,41,42,43]. Due to the varying trends of different active components, it will inevitably affect the proportions of various therapeutic compounds in Astragali Radix extracts, thereby altering the effectiveness of the extracts as medicines and potentially leading to unexpected clinical results. From this perspective, the use of chlormequat may have disadvantages. However, on a positive note, the accumulation of active constituents in Astragali Radix can greatly enhance the efficiency of isolating individual compounds, thereby reducing production costs. In further in-depth research, it is possible to strategically enhance the accumulation of target compounds, designing high-yielding and health-promoting specialized metabolites suitable for commercial use, thus providing specific pharmaceutical compounds.

## 3. Materials and Methods

### 3.1. Chemicals and Reagents

The 50% chlormequat aqueous solution was obtained from Sichuan Guoguang Agrochemical Co., Ltd. (Chengdu, China). The standard compound of chlormequat chloride (98.0%) was purchased from Agro-Environmental Protection Institute (Tianjin, China). Thirteen standard compounds (with purities all over 98.0% confirmed by HPLC-UV analysis), including astragaloside IV, astragaloside I, astragaloside II, astragaloside III, isoastragaloside II, isoastragaloside IV, cycloastragenol, methylnissolin-3-*O*-glucoside, astraisoflavan-7-*O*-β-d-glucoside, formononetin, ononin, calycosin, and calycosin-7-glucoside, were provided by Chengdu Must Bio-technology Co. Ltd. (Chengdu, China). HPLC-grade acetonitrile and methanol were sourced from Fisher Scientific (Fair Lawn, NJ, USA). Other reagents and chemicals were purchased from Sinopharm Chemical Regent Beijing Co., Ltd. (Beijing, China). Ultrapure water intended for HPLC-MS/MS analysis was obtained from a Milli-Q system (Merck Millipore, Billerica, MA, USA).

### 3.2. Plant Materials and Chlormequat Treatment

Biennial plants of *Astragalus mongholicus* were cultivated in experiment base in Sigou Town (Minxian County, Gansu Province). The plant morphology of aerial part and rhizome are shown in Figure 1. The plant density was about 40 plants per m^2^. In July 2020, the land was randomly divided into several experimental districts with an area of about 30 m^2^. Chlormequat aqueous solution (50%) was diluted with water into 10, 1, and 0.1 g/L solutions separately. These different concentrations were uniformly sprayed onto the leaves of the plants using small sprayers. Each concentration was applied in three randomly selected experimental plots, with a second round of spraying done after a one-week interval. The control group of plants did not receive any treatment. Harvesting of Astragali Radix was conducted in November 2020, four months after the chlormequat spraying. The harvested Astragali Radix samples were dried, pulverized, and homogenized before being sealed in Ziplock bags. These samples were then stored at 4 °C until the time of analysis. The identity of the *Astragalus mongholicus* plants was confirmed by Professor Xiaojun Ma, and voucher specimens of both the plant (VS-P-2020-11) and the samples (VS-S-2020-185) were deposited at the Institute of Medicinal Plant Development, Chinese Academy of Medical Sciences, and Peking Union Medical College (Beijing, China).

### 3.3. Instruments

Metabolite identification was conducted on an Acquity UPLC H-Class system coupled with a XevoG2-S QTof™ mass spectrometer (Waters, Milford, MA, USA). Quantitative analysis was carried out using a Shimadzu ultra-fast liquid chromatography (UFLC) system (Shimadzu Corporation, Kyoto, Japan) coupled to an Applied Biosystems Sciex 5500 QTRAP^®^ mass spectrometer system (AB SCIEX, Foster City, CA, USA). Sample preparation and mobile phase preparation were carried out using various equipment: a KQ-400DE type ultrasonic cleaning machine from Kunshan Ultrasonic Instrument Co., Ltd. (Kunshan, China), a Milli-Q purification system from Millipore (Bedford, MA, USA), a GZX-9070MBE type electric blast drying oven from Shanghai Boxun Industrial Co., Ltd. Medical Equipment Factory (Shanghai, China), and BT25S 1/100,000 electronic analytical balance and BS210S 1/10,000 electronic analytical balance from Sartorius Scientific Instrument (Beijing) Co., Ltd. (Beijing, China).

### 3.4. Standard and Sample Solution Preparation

Stock solutions of astragaloside IV, astragaloside I, astragaloside II, astragaloside III, isoastragaloside II, isoastragaloside IV, cycloastragenol, methylnissolin-3-*O*-glucoside, astraisoflavan-7-*O*-β-d-glucoside, formononetin, ononin, calycosin, and calycosin-7-glucoside were individually prepared at a concentration of 1 mg/mL in methanol. These stock solutions of standards were then further diluted using a mixed solvent of methanol and water (80:20, *v*/*v*) to create mixed standard working solutions. All prepared solutions were stored in a refrigerator at 4 °C prior to analysis. Six different concentrations of mixed standard were analyzed, and subsequent calibration curves were generated by plotting the peak area against the concentration of the target analytes.

For the residue analysis of chlormequat, a portion of homogenized Astragali Radix powder (1.0 g) was carefully weighed and transferred to a 50 mL centrifuge tube. It was then mixed with 10 mL of acetonitrile containing 1% formic acid for extraction. The mixture was vortexed for 1 min and subsequently subjected to ultrasonic extraction at 40 kHz for 10 min. After extraction, the samples were centrifuged at 10,000× *g* for 5 min, and the resulting supernatant was diluted once with ultrapure water. Following another 1-min vortex, the sample solution was filtered through a 0.22-μm syringe nylon filter and stored at −20 °C until analysis.

For the metabolite analysis of Astragali Radix, the homogenized samples (1.0 g) were precisely weighed and subjected to ultrasonic extraction with 20 mL of a methanol–water mixture (80:20, *v*/*v*) for 30 min. Then, 1.0 mL of the upper layer of the extracted solution was filtered through a 0.22 µm syringe nylon filter before analysis. Regarding the quantitative analysis of thirteen active compounds in Astragali Radix, the homogenized samples (1.0 g) were accurately weighed and underwent ultrasonic extraction with 20 mL of methanol–water mixture (80:20, *v*/*v*) for 30 min. The extraction was repeated once, and the extracts were combined. A volume of 1.0 mL of the extract was pipetted and diluted to 10 mL using methanol–water (80:20, *v*/*v*). The solution was mixed using a vortex for 30 s, and it was then filtered through a 0.22 μm microporous membrane. All samples were stored in a refrigerator at 4 °C prior to analysis.

### 3.5. Metabolic Profiling Analysis of Astragali Radix

Metabolite identification of Astragali Radix was carried out using an Acquity UPLC H-Class system coupled with a XevoG2-S QTof™ mass spectrometer (Waters, Milford, MA, USA). Chromatographic separation was achieved using an ACQUITY UPLC™ HSS T3 column (100 mm × 2.1 mm, 1.8 μm, Waters). The column was maintained at a temperature of 35 °C, and elution was performed at a flow rate of 0.3 mL/min. The mobile phase consisted of acetonitrile (A) and water with 0.1% formic acid (B), following a gradient elution: 90–80% (B) from 0 to 5 min; 80–35% (B) from 5 to 20 min; 35–10% (B) from 20 to 25 min; 10–90% (B) from 25 to 26 min; followed by equilibration using 90% B from 26 to 30 min.

Mass spectrometry with electrospray ionization (ESI) was conducted in the positive mode. The mass spectrometer settings were as follows: capillary voltage at 3 kV, sampling cone at 30 V, extraction cone at 4.0 V, source temperature at 100 °C, and desolvation temperature at 300 °C. A collision energy of 15 eV was utilized during the MS acquisition, while 45 eV was employed during the MSE acquisition. The cone gas flow rate was set at 50 L/h. Time-of-flight (TOF) MS scanning covered a mass range of *m*/*z* 50–1200 Da. Data collection was performed using MassLynx V4.1 software.

### 3.6. Quantitative Analysis of Thirteen Main Components in Astragali Radix

#### 3.6.1. UFLC-QTRAP-MS/MS Conditions

Quantitative analysis was performed using a Shimadzu ultra-fast liquid chromatography (UFLC) system (Shimadzu Corporation, Kyoto, Japan) coupled to an Applied Biosystems Sciex 5500 QTRAP^®^ mass spectrometer system (AB SCIEX, Foster City, CA, USA). Chromatographic separation was achieved using an ACQUITY UPLC™ HSS T3 column (100 mm × 2.1 mm, 1.8 μm, Waters). The mobile phase consisted of water with acetonitrile (A) and 0.1% formic acid (B), with a gradient elution: 90–70% (B) from 0 to 2 min; 70–35% (B) from 2 to 10 min; 35–15% (B) from 10 to 11 min; 15–90% (B) from 11 to 12 min; followed by elution with 90% B for 3 min. The column was operated at a flow rate of 0.3 mL/min, and the injection volume was 2.0 μL.

Mass spectrometry detection was conducted using the electrospray ionization (ESI) source in positive mode. Curtain gas (CUR), nebulizer gas (GS1), and auxiliary gas (GS2) were set at 35, 50, and 50 psi, respectively. The ion spray voltage (IS) was set to 5500 V, and the source temperature was maintained at 550 °C. Multiple reaction monitoring (MRM) mode was employed for quantitation, with a dwell time of 50 ms for each MRM transition. Applied Biosystems Analyst software (version 1.6) was used for controlling the UFLC-QTRAP-MS/MS system, as well as for data acquisition and processing.

#### 3.6.2. Method Validation

Following the guidelines of the International Conference on Harmonization (ICH) for analytical method validation [44], the validation of the method encompassed various aspects including linearity, selectivity, sensitivity, precision (both intra-day and inter-day variability), stability, and accuracy. Calibration curves were established using the relationship between the analyte concentration (X) and the corresponding peak area (Y). Each calibration curve was created with a minimum of six concentration levels, and these were analyzed in triplicate. The limits of detection (LOD) and quantification (LOQ) for each analyte were determined at signal-to-noise (S/N) ratios of approximately 3 and 10, respectively. To assess precision, the method was tested with repeated analyses (n = 6) of standard samples within a single day (intra-day variation) and over three consecutive days (inter-day variation). Accuracy was evaluated by introducing the targeted analytes at three different concentrations into previously analyzed samples. Stability was examined by analyzing the sample solution at room temperature. The solution was assessed in triplicate every 12 h over a span of 2 days.

## 4. Conclusions

The cultivation process of *Astragalus mongholicus* involving the use of chlormequat has led to higher residual levels in Astragali Radix. Furthermore, the residual amount shows a positive correlation with the applied concentration. Through metabolomics methods and quantitative analysis, it has been discovered that different concentrations of chlormequat have brought about significant changes in the specialized metabolites of Astragali Radix. This demonstrates that the chlormequat has distinct regulatory effects on specialized metabolites such as saponins and flavonoids. Most saponin components have seen an increase in content, while certain flavonoid components have experienced a decrease. This has led to changes in the proportions of various active compounds in Astragali Radix. Consequently, it raises questions about whether the residue of chlormequat and the alteration of specialized metabolites in Astragali Radix could impact the efficacy and safety of clinical applications in the later stages. Therefore, the decision to utilize chlormequat and the manner in which it should be employed scientifically warrant further investigation and attention in subsequent studies.

## Figures and Tables

**Table 1:** The detailed information of identified compounds in Astragali Radix.

Compound No.	Rt	*m*/*z*	Formula	Fragment	Identification	Classification	Regulation
**1**	6.71	679.5082	C_38_H_63_O_10_	661.4999; 359.2351; 340.2571; 114.0903	3β-acetoxy-9β, 19-cyclolanost-24E-ene-1α, 16β-diol-27-*O*-β-d-glucopyranoside	Saponins	
**2**	10.37	785.4671	C_41_H_69_O_14_	455.3521; 437.3397; 419.3304; 285.0744; 270.0528	Astragaloside III		Up
**3**	10.95	947.517	C_47_H_79_O_19_	774.3220; 473.3628; 437.3397	Astragaloside V		Up
**4**	11.44	785.4645	C_41_H_69_O_14_	455.3477; 437.3397; 419.3304; 143.1061	Astragaloside IV		
**5**	11.75	785.4687	C_41_H_69_O_14_	455.3628; 437.3397; 419.3304	Isoastragaloside IV		
**6**	12.5	827.4783	C_43_H_71_O_15_	809.4662; 455.3628; 419.3304; 175.0590	Astragaloside II		Up
**7**	12.99	827.4793	C_43_H_71_O_15_	809.4662; 455.3628; 437.3397; 419.3304; 143.1061	Isoastragaloside II		Up
**8**	13.38	827.478	C_43_H_71_O_15_	629.4077; 611.3973; 437.3397; 419.3304; 143.1061	Cyclocephaloside II		
**9**	13.88	751.4265	C_40_H_63_O_13_	733.4155; 715.4075; 419.3304; 261.0570; 157.0492	Huangqiyenin G		Up
**10**	14.32	869.4891	C_45_H_73_O_16_	851.4815; 689.4276; 671.4146; 455.3628; 437.3397; 419.3304; 217.0688; 157.0492; 143.1061	Astragaloside I		Up
**11**	14.71	869.4876	C_45_H_73_O_16_	851.4815; 689.4276; 671.4146; 455.3628; 437.3397; 419.3304; 376.2594; 217.0688; 157.0492	Isoastragaloside I		
**12**	14.74	943.5263	C_48_H_79_O_18_	617.4073; 599.3953; 441.3712; 423.3624	Soyasaponin I		
**13**	14.97	491.374	C_30_H_5_1O_5_	491.3740; 473.3628; 455.3628	Cycloastragenol		
**14**	15.39	955.4885	C_48_H_75_O_19_	937.4789; 775.4269; 455.3628; 243.0472; 157.0492; 143.1061	Astroolesaponin D		Up
**15**	5.43	447.1276	C_22_H_23_O_10_	447.1286; 285.0744; 270.0494	Calycosin-7-glucoside	Flavonoids	
**16**	7.99	431.1326	C_22_H_23_O_9_	431.1317; 269.0785	Ononin		
**17**	8.45	463.1571	C_23_H_27_O_10_	301.1059; 167.0686	Methylnissolin-3-*O*-glucoside		Down
**18**	9.04	465.1754	C_23_H_29_O_10_	429.1541; 302.1151; 167.0686	Astraisoflavan-7-*O*-β-d-glucoside		Down
**19**	10.33	285.0752	C_16_H_13_O_5_	270.052; 253.0495; 225.0543; 197.0592; 137.0234	Calycosin		
**20**	13.3	269.0801	C_16_H_13_O_4_	253.0495; 237.0525; 226.0623; 213.0898; 197.0592	Formononetin		
**21**	7.46	533.1271	C_25_H_25_O_13_	285.0744	Calycosin-7-glucoside-6″-malonate		Down
**22**	8.97	549.1232	C_25_H_25_O_14_	301.1059; 270.0528	Methylnissolin-3-*O*-glucoside-6″-malonate		Down
**23**	10.1	301.1059	C_17_H_17_O_5_	204.0861; 167.0686	Isomer of medicarpin		Down
**24**	12.77	301.1059	C_16_H_13_O_6_	285.0744; 167.0686; 152.0485; 134.0388	Medicarpin		
**25**	9.6	517.1331	C_25_H_25_O_12_	269.0785	Formononetin-7-*O*-β-d-glucoside-6″-malonate		
**26**	9.81	549.1595	C_25_H_25_O_14_	371.0977; 301.1059; 167.0686	Astrapterocarpanglucoside-6″--malonate		Down

**Table 2:** The contents of thirteen compounds in Astragali Radix sample (μg/g, mean ± SD, n = 3).

Compounds	Treatment Groups			
	CK	T1	T2	T3
Astragaloside I (**10**)	1664.77 ± 128.69	1907.67 ± 61.20	2106.41 ± 166.72	2760.05 ± 490.77
Astragaloside II (**6**)	375.33 ± 34.27	380.84 ± 54.53	396.20 ± 71.63	482.21 ± 92.36
Astragaloside III (**2**)	16.04 ± 0.56	15.87 ± 0.20	16.47 ± 0.33	15.50 ± 0.15
Astragaloside IV (**4**)	55.83 ± 3.13	64.22 ± 8.67	76.80 ± 5.90	61.51 ± 3.41
Isoastragaloside II (**7**)	129.58 ± 5.79	135.84 ± 27.74	160.97 ± 5.60	201.66 ± 12.30
Isoastragaloside IV (**5**)	94.00 ± 5.42	131.95 ± 21.39	136.62 ± 25.60	131.04 ± 13.51
Cycloastragenol (**13**)	8.19 ± 0.14	8.87 ± 0.05	8.75 ± 0.87	8.87 ± 1.08
Methylnissolin 3-*O*-glucoside (**17**)	153.62 ± 27.63	89.58 ± 27.11	116.98 ± 9.37	119.55 ± 13.98
Astraisoflavan-7-*O*-β-d-glucoside (**18**)	67.48 ± 5.38	43.89 ± 6.69	53.25 ± 14.05	35.24 ± 0.89
Formononetin (**20**)	66.57 ± 0.97	62.70 ± 5.64	91.68 ± 2.16	113.22 ± 21.71
Ononin (**16**)	99.41 ± 9.64	77.88 ± 14.29	117.79 ± 14.91	102.07 ± 16.68
Calycosin (**19**)	101.95 ± 3.41	117.17 ± 15.73	115.85 ± 12.88	113.24 ± 8.31
Calycosin-7-glucoside (**15**)	271.93 ± 14.00	213.12 ± 24.96	294.81 ± 28.58	245.15 ± 26.33

## Data Availability

Not applicable.

## Supplementary Materials

The following supporting information can be downloaded at: https://www.mdpi.com/article/10.3390/molecules28196754/s1, Figure S1: The TIC chromatography of different treatment groups of Astragali Radix. a, represents the control group; b, the chlormequat concentration was 0.1 g/L; c, the chlormequat concentration was 1.0 g/L, and d indicated the chlormequat concentration was 10.0 g/L; Table S1: Chlormequat residue levels in different treatment groups of Astragali Radix; Table S2: The identified metabolites and their response intensities in different treatment samples; Table S3: The optimized MS parameters for the targeted analytes; Table S4: Method validation results including the linearity, limit of detection (LOD) and limit of quantification (LOQ), precision (relative standard deviation, RSD, %), recovery (%), and stability (RSD, %).
