# Supplementary material for: Residue of Chlormequat and Regulatory Effects on the Specialized Metabolites of Astragali Radix
Source: Molecules. 2023 Sep 22;28(19):6754. doi: 10.3390/molecules28196754 (PMC10574182; doi:10.3390/molecules28196754)
Supplement: Supplementary file 1 [file molecules-28-06754-s001.zip › molecules-2590603-supplementary.pdf]

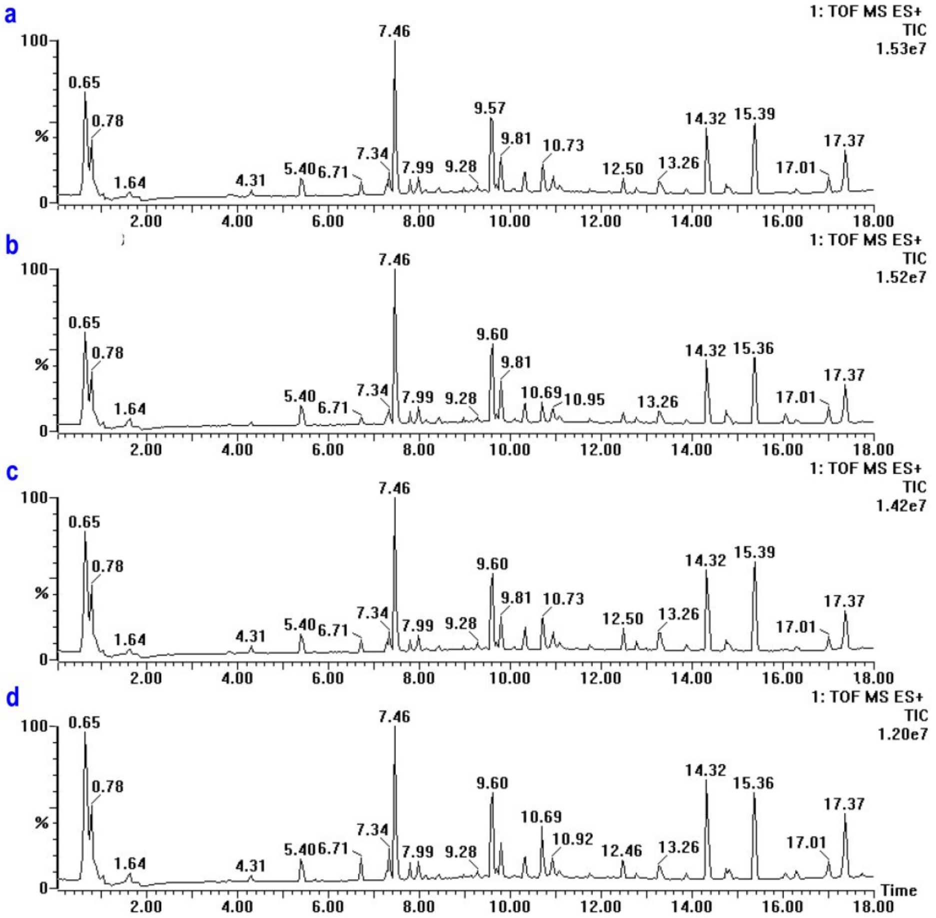

Supplementary Figure S1. The TIC chromatography of different treatment groups of Astragali Radix. a, represents the control group; b, the chlormequat concentration was 0.1 g/L; c, the chlormequat concentration was 1.0 g/L, and d indicated the chlormequat concentration was 10.0 g/L.

Supplementary Table S1. Chlormequat residue levels in different treatment groups of Astragali Radix.

Concentrations of chlormequat	Samples	Residue Level (μg/kg)
	CK-1	25.02
—	CK-2	24.25
	CK-3	19.18
	T1-1	163.80
0.1 g/L	T1-2	179.40
	T1-3	218.95
	T2-1	690.56
1.0 g/L	T2-2	458.81
	T2-3	553.22
	T3-1	5608.68
10.0 g/L	T3-2	5099.12
	T3-3	5501.23

Supplementary Table S2. The dentified metabolites and their response intensities in different treatment samples.

Compound	CK1	CK2	CK3	T1-1	T1-2	T1-3	T2-1	T2-2	T2-3	T3-1	T3-2	T3-3
	CK	CK	CK	T1	T1	T1	T2	T2	T2	T3	T3	T3
6.71_679.5082	2314	3177	4071	3884	6118	5591	11276	5555	7655	1869	3431	8215
10.37_785.4671	2	1	2	2	2	2	4	2	2	2	1	1
10.95_954.1771	1430	1495	1414	1811	1411	1630	1854	1909	1646	2016	2023	1930
11.44_785.4645	14	16	13	15	19	18	18	17	18	18	19	16
11.75_785.4688	365	452	441	656	694	446	632	752	826	894	491	618
12.50_826.4710	3487	4042	3830	3596	4079	3957	6063	5668	5717	5851	5799	6000
12.99_827.4793	60	65	85	59	68	69	82	111	105	101	100	111
13.38_827.478	49	16	35	16	14	21	13	16	15	19	11	17
13.88_751.4265	145	125	121	206	162	207	213	303	162	282	223	282
14.32_868.4819	15151	19426	16646	19133	17569	17483	24375	23005	23761	24112	23777	23381
14.71_869.4907	30	41	44	47	40	29	48	58	31	54	29	64
14.74_943.5263	14085	16321	13826	17635	13737	12513	13346	14343	16390	15701	15228	14605
14.97_491.374	607	420	394	652	492	582	744	557	477	457	443	406
15.39_955.9906	6186	6565	6790	10589	8233	9647	10472	13245	9389	10668	10261	10774
5.40_447.1276	13680	16347	11113	11978	10544	9949	10526	9788	12775	9067	9280	10030
7.46_532.1206	111329	117449	92992	103915	99254	86564	86811	85279	94558	74057	75395	78052
7.99_431.1326	9205	11171	6979	7697	7936	6650	7348	6299	7642	5952	6126	6876
8.45_463.1571	2971	3137	2192	2023	1830	1641	1252	1476	2027	807	987	1405
8.97_549.1232	3680	3576	2934	2781	2727	2369	2167	2178	2710	1795	2069	1818
9.04_464.1681	15	18	36	5	7	8	10	5	11	9	4	10
9.60_516.1258	79803	86787	63041	66944	72707	59061	63614	57944	63573	52313	54779	58488
9.81_548.1522	24265	24940	19661	18309	16030	14104	10055	12586	15721	8203	9598	12900

10.10_301.1059	154	159	138	99	87	68	31	48	74	22	26	52
10.33_285.0752	10242	12275	8095	9211	8755	8806	12528	11023	13030	11573	9460	8046
12.77_301.1059	3692	3958	2457	2814	2716	2551	3335	4119	4558	2764	2338	2409
13.30_269.0801	9087	12224	7083	8557	10613	7485	12881	12083	13172	21282	9632	7753

Supplementary Table S3. The optimized MS parameters for the targeted analytes.

No.	Compound	Q1	Precursor Ion (m/z)	Product Ions (m/z)	CE (V)	Ionization Mode	Rt（min）
1	Astragaloside IV	785.6	120	143.1*/437.3.0	30/20	ESI+	6.69
2	Astragaloside I	869.5	120	143.2*/437.3	30/20	ESI+	8.8
3	Astragaloside II	827.5	120	143.0*/437.5	30/20	ESI+	7.44
4	Astragaloside III	785.6	120	143.1*/437.3	30/20	ESI+	5.87
5	Cycloastragenol	491.5	90	143.0*/437	20/15	ESI+	9.48
6	Isoastragaloside II	827.5	120	143.0*/437.5	30/20	ESI+	7.83
7	Isoastragaloside IV	785.6	120	143.1*/437.3	30/20	ESI+	6.91
8	Methylnissolin 3-O-glucoside	463.4	90	167.0*/301	35/15	ESI+	5.1
9	Astraisoflavan-7-O-β-D-glucoside	465.2	90	123.0*/167.1	30/30	ESI+	5.34
10	Formononetin	269.1	120	253.1*/226.1	40/40	ESI+	8.29
11	Ononin	431.2	180	269.1*/254	30/55	ESI+	4.87
12	Calycosin	285.1	120	270.1*/137.1	30/20	ESI+	6.26
13	Calycosin-7-glucoside	447.4	180	285.1*/270.1	30/55	ESI+	3.96

Note: * Product Ions used for quantification

Supplementary Table S4. Method validation results including the linearity, limit of detection (LOD) and limit of quantification (LOQ), precision (relative standard deviation, RSD, %), recovery (%), and stability (RSD, %).

Analytes	Linearity		LOD (ng/mL)	LOQ (ng/mL)	Precision (RSD, %)		Recovery% (RSD, %)			Stability (RSD, %)
	r^2	Range (ng/mL)			Intra-Day	Inter-Day	Low Level	Medium Level	High Level	
Astragaloside IV	0.9998	10-2500	10	30	2.02	2.67	95.28（1.47）	93.45（2.02）	97.34（1.06）	1.24
Astragaloside I	0.9935	100-2500	20	50	1.2	1.33	91.74（0.47）	91.32(1.22)	87.59(3.71)	3.17
Astragaloside II	0.9939	10-2500	20	50	2.38	1.86	82.55（2.12）	81.54(0.96)	101.82(1.89)	1.98
Astragaloside III	0.9925	10-2500	5	10	1.82	2.17	102.90（1.28）	103.29(0.96)	103.47(2.73)	3.51
Cycloastragenol	0.9996	10-2500	5	10	1.97	3.81	110.49（0.34）	97.03(1.40)	97.64(1.48)	1.23
Isoastragaloside II	0.9978	10-2500	20	50	2.65	2.13	101.37（2.36）	94.59（1.38）	88.00(3.38)	3.2
Isoastragaloside IV	0.9925	100-2500	10	30	1.73	2.05	92.40（5.36）	91.40（3.10）	96.15(2.34)	2.43
Methylnissolin 3-O-glucoside	0.9983	10-1000	0.2	0.5	2.6	2.41	100.84（3.01）	101.66（1.55）	101.58(1.55)	4.21
Astraisoflavan-7-O-β-D-glucoside	0.9991	10-2500	2	5	1.91	1.26	82.91（1.16）	96.71（0.67）	98.41(1.05)	1.71
Formononetin	0.9996	10-500	0.2	0.5	1.21	1.38	85.51(4.46)	101.71(1.31)	99.11(1.76)	2.75
Ononin	0.9991	10-500	0.2	0.5	1.85	1.52	97.72(0.56)	90.40(5.22）	95.20（1.64）	3.87
Calycosin	0.9993	10-500	0.2	0.5	1.82	1.25	100.56（5.19）	104.38（1.50）	96.76（1.66）	1.44
Calycosin-7-glucoside	0.9981	10-1000	0.2	0.5	1.01	0.97	90.38（0.74）	92.13（2.30）	98.93（4.44）	0.53
